# Supplementary material for: Investigating the Challenges and Benefits of Engaging in Peer Support via Videoconferencing for People with Spinal Cord Injury
Source: Int J Environ Res Public Health. 2022 Apr 11;19(8):4585. doi: 10.3390/ijerph19084585 (PMC9026552; doi:10.3390/ijerph19084585)
Supplement: Supplementary file 1 [file ijerph-19-04585-s001.zip › ijerph-1654515-supplementary.pdf]

Supplementary File

Survey questions for: Investigating the challenges and benefits of engaging in peer support via videoconferencing for people with spinal cord injury.

1.What is your gender?

Female

Male

Other

Prefer not to say

2. What is your age range?

18 – 30 years of age

30 – 39 years

40 – 49 years

50 – 59 years

60 – 69 years

70 – 79 years

Over 80 years

3. Which of the following best describes your level and type of injury?

High quadriplegia/tetraplegia – significant involvement of upper limbs, fully dependent on carers for self care

Quadriplegia/tetraplegia –involvement of upper limbs, need some/full assistance from carers for self care

Quadriplegia/tetraplegia –involvement of upper limbs, fully independent in self care

Paraplegia – no involvement of upper limbs, require a wheelchair and some assistance with self care

Paraplegia – no involvement of upper limbs, can walk short distances, independent with self care

Paraplegia – no involvement of upper limbs, able to walk, independent with self care

4. What is the highest level of education you attained?

Year 10 secondary school

Year 11 or 12 secondary school

TAFE course

University degree

Higher degree (masters or doctoral level)

5. Which of the following best describes your employment situation?

Employed part time

Employed full time

Student

Retired

Not seeking employment

Self-employed/own business

Tick all that apply.

Other ________________________

6. Who do you live with?

Alone

Parents

Partner/spouse

Other

7.What best describes your housing situation?

Own home with no mortgage

Own home with mortgage

Home owned by someone else (e.g. parents)

Renting

Residential care

Assisted living arrangement (eg paid carers on site)

8. Do you receive funding from any of the following?

Transport accident insurance

Work insurance

NDIS (Australia)

MyAgedCare (Australia)

Other

9. Do you have a paid personal carer?

Yes

No

10. Do you have a therapist that sees you regularly?

Yes

If yes, please specify type ___________________

No

11. On a scale of 1- 3 please select your experience with videoconferencing prior to COVID

1 – No experience

2 – Some experience

3 – A great deal of experience

12. On a scale of 1-5 please select your usual level of comfort with technology

1- Very uncomfortable
2- Somewhat uncomfortable
3- Neutral
4- Somewhat comfortable
5- Very comfortable

13. Please indicate if you have used any of the following platforms or programs:

Skype: Yes/No

Zoom: Yes/No

Google hangouts: Yes/No

Microsoft teams: Yes/No

FaceTime: Yes/No

Facebook Messenger: Yes/No

Whatsapp: Yes/No

Viber: Yes/No

Other _____________

14. How would you rate your experience using videoconferencing for peer support?

1- Very poor
2- Somewhat poor
3- Neutral

4- Somewhat successful

5- Very successful

15. Did you need assistance to get set up?

Yes

No

16. If yes, who helped you?

Family member

Service provider/therapist

Personal care assistant

Other ___________

17. What were the positive aspects of using videoconferencing for peer support?

Enabled me to connect when otherwise I couldn't

Convenient as I didn't have to leave the house

New skill and format of communication I haven't previously tried

Convenient as I could pick the time

Other____

____

18. What were the challenges/barriers of using videoconferencing for peer support?

Wifi/internet connection issues

Needing help to get set up

Cost

Learning new platform/program

Inconsistencies between platforms/programs

Not getting non-verbal feedback from other person

Other

____

____

19. On a scale of 1-5, how did it compare with face to face/in person support?

1- Compared very poorly

2- Compared somewhat poorly

3- Neutral

4- Compared somewhat well

5- Compared very well

20. Did using videoconferencing help you to feel socially connected?

Yes

No

21. Was the person you were getting peer support from a new person or someone you already knew?

Someone new

Someone I knew already

Other ___________

What do you think were the advantages/disadvantages of this?

____

____

22. Are there other comments you would like to make?

____

____
